# When Right Feels Left: Referral of Touch and Ownership between the Hands

**DOI:** 10.1371/journal.pone.0006933

**Published:** 2009-09-09

**Authors:** Valeria I. Petkova, H. Henrik Ehrsson

**Affiliations:** Department of Neuroscience, Karolinska Institutet, Stockholm, Sweden; The University of Western Ontario, Canada

## Abstract

Feeling touch on a body part is paradigmatically considered to require stimulation of tactile afferents from the body part in question, at least in healthy non-synaesthetic individuals. In contrast to this view, we report a perceptual illusion where people experience “phantom touches” on a right rubber hand when they see it brushed simultaneously with brushes applied to their left hand. Such illusory duplication and transfer of touch from the left to the right hand was only elicited when a homologous (i.e., left and right) pair of hands was brushed in synchrony for an extended period of time. This stimulation caused the majority of our participants to perceive the right rubber hand as their own and to sense two distinct touches – one located on the right rubber hand and the other on their left (stimulated) hand. This effect was supported by quantitative subjective reports in the form of questionnaires, behavioral data from a task in which participants pointed to the felt location of their right hand, and physiological evidence obtained by skin conductance responses when threatening the model hand. Our findings suggest that visual information augments subthreshold somatosensory responses in the ipsilateral hemisphere, thus producing a tactile experience from the non-stimulated body part. This finding is important because it reveals a new bilateral multisensory mechanism for tactile perception and limb ownership.

## Introduction

Under normal conditions, humans are highly capable of localizing touch to a particular area of skin being stimulated. Visual information can further guide the localization of touch [1], [2], modify the quality of somatic sensations [3], [4], [5] and improve tactile acuity in healthy individuals [6], [7]. But neither visual nor auditory stimuli have been considered to be able to cause tactile sensations in the absence of physical stimuli activating peripheral tactile receptors in the skin of healthy individuals. Thus multisensory signals are viewed as having only a modulatory role in tactile perception, as they have in unimodal perception more generally [8], [9], [10], [11].

In some neurological cases, however, the boundary between multisensory processes and unimodal perception has been dissolved. In this respect Halligan and colleagues described a patient with hemiparesis after stroke who felt the touch when he merely watched a touch being applied to his paralyzed limb [12]. Similarly, in patients with hands rendered anesthetic by stroke or neurosurgery, touches applied to the intact hand produced tactile sensations in the anesthetic hand [13]. Ramachandran and colleagues reported a similar phenomenon when upper limb amputees saw a mirror image of their intact hand superimposed on their stump, which was hidden from their view behind a mirror [14]. When they saw the ‘missing limb’ being touched in the mirror, they reported feeling touches on their phantom limb [15]. These neurological cases suggest that brain plasticity and central reorganization might up-regulate the processing of tactile signals from the ispilateral intact body half, and that these signals can be combined with visual signals from the impaired limb, resulting in “phantom touch sensations”. Presumably, this happens via plastic changes in the multisensory areas in the posterior parietal cortex [13] that integrate tactile information from the hands [16], [17], [18] with visual information [19], [20], [21], [22], [23], [24].

The sense of touch is intimately linked to the perception of one's own body. A limb that can feel touch is typically experienced as being one's own, as was famously demonstrated in the case of the rubber hand illusion [25]. In this illusion, simultaneous brushing of a rubber hand in full view of the participant, and of the participant's hand, which is out of view behind a screen, produces the illusion that the participant feels the touch of the paintbrush ‘in’ the rubber hand and experiences the dummy hand as his or her own hand [26], [27]. The referral of touch and ownership to the rubber hand only works if certain criteria are satisfied, namely, that: the rubber hand and the real one are touched synchronously [25], [26], [28], the rubber hand is aligned parallel to the hidden real hand [26], [28], [29], [30], the two hands are touched on corresponding sites [31], the rubber hand is of the same laterality as the hidden hand (e.g., right rubber hand and right real hand [28]), and the distance between the hands is less than 35 cm [32]. This indicates that the visuo-tactile integration underlying the phenomenon operates in arm-centered reference frames in near-personal space [27], [29], probably mediated via multisensory neuronal populations in the premotor cortex and posterior parietal cortex [20], [26], [33], [34].

In our laboratory, we recently discovered an unexpected version of the rubber hand illusion that demonstrates an important new role played by homologous limbs for the sense of ownership and tactile perception. We found that healthy participants can experience a “phantom touch” on a right rubber hand that they see being brushed in the absence of any touch delivered to their hidden right hand. This occurs when the *contralateral* left hand is stimulated synchronously at the corresponding homologue's site. This “bimanual transfer of touch” is also associated with the feeling of ownership of the rubber hand. These findings are of fundamental importance because they reveal how multisensory interactions between the hands cause qualitative changes in unimodal tactile perception, and that this has a direct consequence for how we come to experience limbs as part of our own body.

## Methods

### Participants

Thirty healthy naïve participants (mean±s.d. age 25±5 years, 15 females) participated in our first experiment. For the second experiment, a new group of fourteen volunteers was recruited (mean±s.d. age 24±6 years, 8 females). Another group of fourteen volunteers participated in our third experiment (mean±s.d. age 26±9 years, 7 females). Thirteen new participants took part in the fourth experiment (mean±s.d. age 29±7 years, 5 females). All participants gave their written informed consent prior to participating in the relevant experiment. This study was conducted according to the principles expressed in the Declaration of Helsinki. The study was approved by the Institutional Review Board of the Regional Ethics committee of Stockholm and Karolinska hospitals. All participants provided written informed consent for the collection of samples and subsequent analysis.

### Experimental design

The experiments were designed to include three experimental manipulations and to obtain three complementary measures of the illusion (see below for details). We changed the timing of the stimulation on the two hands, hypothesizing that only synchronous stimulation would produce the illusion (Experiments #1, #2, and #3). The orientation of the rubber hand was also varied (Experiment #2) to test the prediction that the right rubber hand has to be aligned with the participant's own hand, i.e. that it has to be placed in an anatomically congruent position. Finally, we studied the effect of the laterality of the hand – right vs. left – to test the hypothesis that the illusion only works for a homologous pair of a left hand and a right (rubber) hand. The combination of subjective (Experiment #1), physiological (Experiment #2) and behavioral (Experiment #3 and #4) measures of the illusion provides robust and corroborative evidence for the illusion.

### Experimental setup

**Figure 1:**
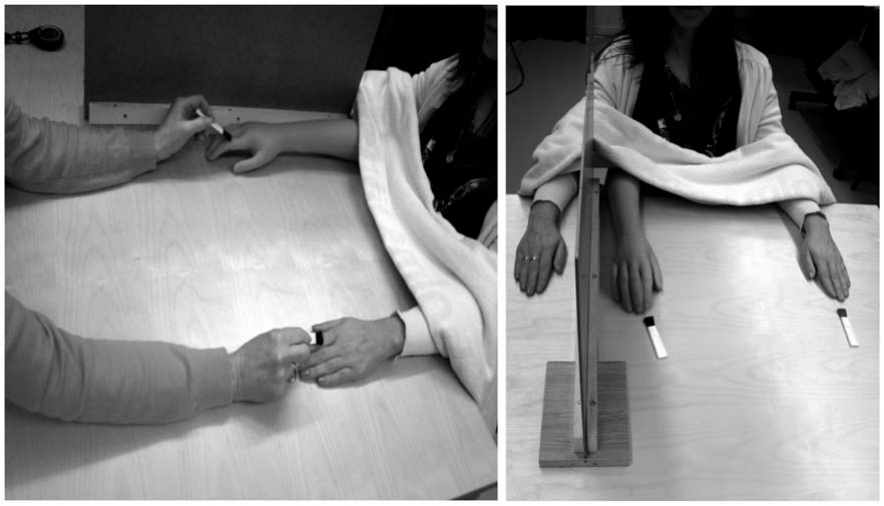
Experimental set-up.

The participants were seated with their arms resting prone on a table as depicted in Figure 1. A life-size right cosmetic hand prosthesis was placed on the table twenty one centimeters to the right of the midline of the participants' body. The real right hand was hidden behind a screen at a distance of twenty centimeters from the rubber hand. The left hand was placed in full view twenty one centimeters to the left of the midline of the body. A towel was laid over the proximal ends of the arms to cover the gap between the rubber arm and the person's body. The set up, thus, created the visual impression that the participants had placed both of their hands on the table parallel to one another (Figure 1). All participants were instructed to look at the rubber hand. Two identical brushes were used to stroke the left real and the right rubber hand either synchronously (corresponding to the illusion condition used in all experiments) or asynchronously (providing the control condition for Experiments #1, #2, and #3). The touches were delivered to the corresponding parts of the index and middle fingers of the right rubber hand and left real hand. An irregular, but synchronous, rhythm of brushing was chosen to enhance the illusion since this mode of stimulation is known to maximize the traditional rubber hand illusion (unpublished observations). The brushing in the asynchronous condition was in an irregular and alternating pattern. The participants were explicitly instructed not to move their right hand behind the occluding screen.

### Questionnaire data (Experiment #1)

Our first experiment consisted of two sessions, one of synchronous and one of asynchronous brushing of the two visible hands (i.e. the left real hand and the right rubber hand). Each session lasted five minutes. Half of the participants started with the synchronous and the other half started with the asynchronous condition. At the end of each session, the participants were asked to fill out a short questionnaire, which consisted of nine statements about the experiences they might have had during the stimulation. Four statements (Q1–Q4) were designed to capture different aspects of the illusory perception related to the sensation of touches on the rubber hand and the feeling of ownership of that hand. One statement (Q5) was constructed to explore possible sensations in the real right hand induced by the visuo-tactile conflict, as suggested by the results of a previous study [35] and pilot experiments. Statements Q6–Q9 served as control questions for task compliance and susceptibility effects (see Table 1). The participants were asked to rate their level of agreement with the statements on a seven-point Likert scale with a range from “**+**3” (agree very strongly) to “−3” (disagree very strongly) where “0” corresponded to neither agreeing nor disagreeing.

**Table 1:** The statements presented to the participants after 5 minutes of synchronous vs. asynchronous brushing.

Statements	Yes	Uncertain	No
Q1: I felt as if the rubber hand was my hand	16	3	11
Q2: It seemed as though the touch I felt was caused by the paintbrush touching the rubber hand	21	1	8
Q3: It seemed as if I was feeling the touch of the paintbrush on the rubber hand	16	4	10
Q4: I could sense two touches, both on my (real) left hand and on the right rubber hand	16	2	12
Q5: I had (weak) sensations of tingling/prickling/tickling or touch in my real right hand (behind the screen)	11	1	18
Q6: It seemed as if I had two left hands or arms	8	3	19
Q7: It seemed as if the touch I was feeling came from somewhere between my own left hand and the rubber hand	10	3	17
Q8: It felt as if my (real right) hand were turning ‘rubbery’	7	4	19
Q9: The rubber hand began to resemble my own (real right) hand, in terms of shape, skin tone, freckles or some other visual feature	21	1	8

The participants were asked to rate the degree of their agreement vs. disagreement with those statements using the following scale: ‘+1’ = ‘I agree’; ‘+2’ = ‘I agree strongly’; ‘+3’ = ‘I agree very strongly’; ‘0’ = ‘I am not sure’, ‘−1’ = ‘I disagree’; ‘−2’ = ‘I disagree strongly’; ‘−3’ = ‘I disagree very strongly’. The last three columns of the table represent the number of people rating these statements with ≥+1 (yes), 0 (uncertain), or ≤−1(no).

### Physiological recordings (Experiment #2)

In the second experiment, we measured the skin conductance response following the simulation of physical injury to the rubber hand. This experiment was included to provide objective physiological evidence for the illusion. Previous work has demonstrated a relationship between the feeling of ownership of a rubber hand and the anxiety experienced when this hand is being subjected to physical threats [36], [37]. The anxiety triggered by physical threats leads to changes in skin sweating that lead to changes in skin conductance. We included three conditions: the synchronous or asynchronous stimulation conditions from Experiment #1; and a third condition, where the rubber hand was rotated 180 degrees and synchronous stimulation was applied. The latter experimental manipulation is known to reduce the traditional rubber hand illusion [26]. We included this condition to control for possible association learning effects induced by a period of synchronized visual and tactile stimuli.

All three conditions were repeated three times in an order that was balanced across the participants. At the end of each session, a needle was stabbed into the rubber hand and the skin conductance response (SCR) was measured with two Ag-AgCl reusable electrodes attached to the middle and index fingers of the right hand, hidden behind the screen. We used Signa electrode gel (Parker Laboratories, INC., New Jersey, USA). The data were registered with a Biopac System MP150 (100 samples per second) and processed with the Biopac software Acqknowledge for Windows ACK100W. The participant wore the electrodes for a few minutes before starting the recording. The parameters of the recording were as follows: The gain switch was set to 5 µmho/V and the CAL2 Scale Value was set to 5. The timing of the stabbing events was indicated in the raw data files during the recordings by the experimenter, by pressing a key. A one-way repeated ANOVA was used to test for statistical differences in the SCRs for the three conditions. The SCR was identified as the peak in the conductance that occurs up to 5 seconds after the onset of the threat stimuli. The amplitude of the SCR was measured as the difference between the minimal and maximal values of the response identified in this time-window. We calculated the average of the all responses including the trials where no response was apparent, thus, analysing the magnitude of the SRC [38]. Participants who did not show a reliable threat-evoked SCR (‘null responders’), i.e. had zero responses in more than two-thirds of the trials, were excluded from the analysis.

### Proprioceptive drift measure (Experiments #3 and #4)

In the traditional rubber hand illusion, the feeling of touch on the rubber hand is associated with a drift in the perceived location of the hand towards the location of the rubber hand [25], [28], [39], with both hands having the same handedness. In our third experiment we wanted to determine whether the present illusion of the transfer of touch from one hand to the other was associated with changes in proprioception. This would also provide objective behavioral evidence that the rubber hand is perceived as one's own hand. In this experiment, the participants were exposed to periods of three minutes of synchronous and asynchronous brushing of the left real hand and the right rubber hand (as in Experiment #1).

In a fourth experiment we used this proprioceptive drift measure (see Results) to test the hypothesis that in our set-up the bilateral illusion requires a homologous pair of limbs, i.e. that the effect requires a pair of right and left hands. Thus, as a control condition, we replaced the right rubber hand with a left one and brushed the real left hand and the left rubber hand simultaneously.

In both experiments (#3 and #4), the two conditions were repeated three times in a balanced order across participants. Between each brushing session there was a break of one minute. Directly before and directly after each period of brushing, the participants were asked to close their eyes and indicate the position of their right index finger by pointing with their left hand. Before making this response, the experimenter positioned a ruler 31 centimeters above the table 49 centimeters in front of the participant's body. The experimenter placed the participant's left index finger at the starting point of the ruler, which was just in front of the body midline, and asked him or her to move that finger briskly along the ruler and stop until it was immediately above where he or she felt the right hand to be located. We computed the differences in pointing error (towards the rubber hand) between the measurements made before and after each period of stimulation. The average of the difference values was compared between the two conditions using paired t-tests.

### Statistical analyses

In Experiment #1 we compared the illusion questions to the control questions, in the synchronous and asynchronous conditions, respectively, using a 2×2 ANOVA with the factors Condition (Synchronous, Asynchronous) and Question type (Illusion, Control). Out planned comparison was the interaction between Condition and Question type, i.e. a greater difference between the illusion and control questions during the synchronous stimulation than during the asynchronous stimulation.

In Experiment #1 we also analyzed the correlations of scores on the illusion questions related to feeling touch on the rubber hand and the feeling of limb ownership. In the traditional rubber hand illusion it is well known that these perceptual experiences are tightly coupled (Makin et al. 2008). On the basis on our observations from pilot experiments we predicted that a similar tight correlation should be observed between the experiences of phantom touches and ownership of the model hand in the present set-up.

In Experiment #2 we predicted greater skin conductance responses in the illusion condition than in each of the two control conditions. Thus first we used one-way ANOVA to test for an effect of condition on the SCR. Then we conducted two planned comparisons between the illusion condition to the two control conditions, respectively (corrected for multiple comparisons).

In Experiment #3 we predicated greater proprioceptive drift towards the rubber hand in the illusion condition than in the control conditions. In Experiment #4 we predicted that the proprioceptive drift towards the rubber hand will be observed only when a right rubber hand is brushed in synchrony with the left real hand. We predicted that the effect will be abolished when the right rubber hand is replaced by a left rubber hand. In both experiments we used t-tests to compare the two conditions.

The reader should note that in all our experiments we have used the more conservative two-tailed statistical tests even in the case of planned comparisons with one-tailed predictions. We have used Kolmogorov-Smirnov test to check the parametric assumptions and in cases of violations we have used non-parametric statistical tests, as indicated in the Results section. Apart from the correlation analysis in Experiment #1 in which we set alpha to 2.5% due to multiple comparison between Q1, Q3, and Q4, we set alpha to 5% in all remaining tests.

## Results

### Questionnaire data (Experiment #1)

Sixteen out of the thirty participants (53%) felt as though the rubber hand was their real hand (ratings on statement Q1 of ≥+1) when it was brushed for a prolonged time in synchrony with their left hand (Table 1). Similarity, sixteen participants (53%) reported the sensation of two distinct touches: one on the right rubber hand and the other on the real left hand.

The rating scores were significantly greater on the illusion questions than on the control questions, and this effect was significantly greater after a period of synchronous stimulation as we had predicted. Statistically, we could demonstrate this effect using a two-way 2×2 ANOVA on ranks. Specifically, we obtained significant differences between the levels of the factors “Condition” (synchronous, asynchronous) (N = 30, p<.001, F(1, 29) = 25.367, two-way 2×2 ANOVA on ranks), “Question type” (illusion, control) (N = 30, p = .039, F(1, 29) = 4.674, two-way 2×2 ANOVA on ranks), and crucially, a significant interaction between the two factors (N = 30, p = .035, F(1, 29) = 4.892, two-way 2×2 ANOVA on ranks).

**Figure 2:**
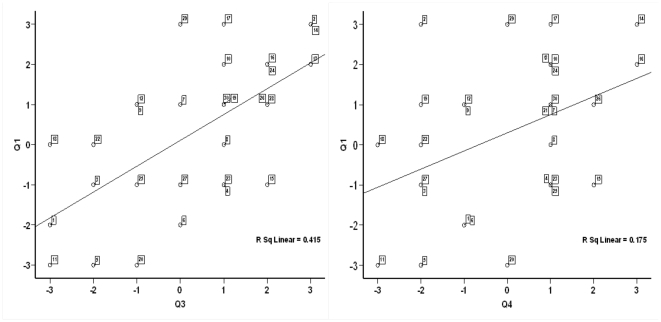
Scatter plots of the correlation between Q1 (“I felt as if the rubber hand was my hand”) and Q3 (“It seemed as if I was feeling the touch of the paintbrush on the rubber hand”) (panel A). Panel B shows the correlation between Q1 (“I felt as if the rubber hand was my hand”) and Q4 (“I could sense two touches both on my (real) left hand and on the right rubber hand”). The correlations between the two pairs of questions were significant (p<.025); for details see the Results sections.

As we predicted there was a significant correlation between experiencing duplication of touch and feeling ownership of the rubber hand (N = 30, p = .021, r = .418, two-tailed Pearson correlation), i.e. a correlation was observed between the ratings of Q1 (“I felt as if the rubber hand was my hand”) and Q4 (“I could sense two touches, both on my (real) left hand and on the right rubber hand”). A highly significant correlation was also observed between the ratings of Q1 (“I felt as if the rubber hand was my hand”) and Q3 (“It seemed as if I was feeling the touch of the paintbrush on the rubber hand”) (N = 30, p<.001, r = .644, two-tailed Pearson correlation) (Figure 2). It was important to analyze the correlations between the different illusion questions because strong correlations would imply that our objective tests for ownership (see below) would provide evidence for experiencing “phantom touches” on the rubber hand.

In two additional pilot experiments we measured how long it took before the onset of the illusory perception of touch: we found that it takes more than one minute of synchronous stimulation. In these pilot tests, we also observed that simply brushing the rubber hand for five minutes without simultaneous brushing of the contralateral real hand does not elicit the illusion. In other words, just seeing the rubber hand brushed does not produce the referral of tactile sensations.

### Physiological recordings (Experiment #2)

**Figure 3:**
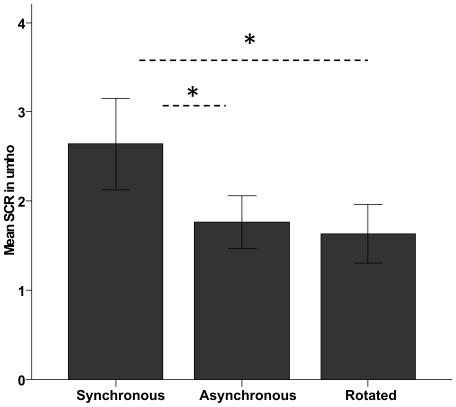
Mean skin conductance responses after threads towards the rubber hand in the three conditions. Error bars indicate standard errors.

In line with our hypothesis people displayed greater skin conductance responses when we stabbed the rubber hand with the needle after the illusion condition than they did under the control conditions. There was a significant effect of condition (synchronous brushing, asynchronous brushing, and synchronous brushing of the rotated rubber hand) in the stabbing-evoked SCR (N = 14, p = 0.028, F(2, 26) = 4.138, one-way repeated measures ANOVA) (Figure 3). We used the Student-Newman-Keuls Method for pair-wise multiple comparison between the different conditions, which yielded significant results for the comparison between the illusion condition and each of the two control conditions (N = 14, p = 0.035 and p = 0.030, respectively), and a non-significant result for the comparison between the two control conditions (N = 14, p = 0.729).

### Proprioceptive drift measure (Experiments #3 and #4)

**Figure 4:**
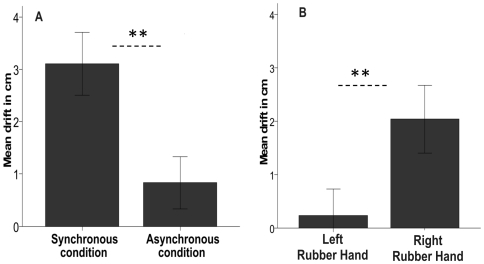
Mean pointing error towards the right rubber hand when the participant was asked to point to his or her own right hand. Panel A shows the pointing error when the rubber hand was brushed in synchrony vs. asynchrony with the left hand of the participant. Panel B shows the difference in the pointing error towards either a left or a right rubber hand when this was brushed in synchrony with the left hand of the participant. Error bars indicate standard errors.

Experiment #3 demonstrated that the illusion was associated with a drift in the perceived location of the right hand towards the rubber hand (Figure 4a). The mispointing towards the rubber hand was significantly greater (3.00±2.25 cm; corresponding to 15.5% of distance between the hands) after the synchronous condition than after the asynchronous one (0.80±1.87 cm; 4%) (N = 14, p = .012, two-tailed t-test).

In our fourth experiment we found that the mispointing in the direction of the rubber hand requires that a rubber hand be used that is from the same laterality as that of the real hand hidden from view, that is, the illusion does not occur when the right rubber hand is replaced with its left counterpart. The proprioceptive drift was significantly greater after a period of synchronous brushing of the right rubber hand (2.04±2.28 cm; 10.2%) than after an equivalent period of stimulation using the left rubber hand (0.23±1.79 cm; 1.15%) (N = 13, p = 0.01, two-tailed t-test) (Figure 4b).

## Discussion

We have reported a perceptual illusion in which touches applied to a participant's left hand are sensed on a right rubber hand when both hands are brushed synchronously. For this phenomenon to occur, the rubber hand had to be a right hand, it had to be oriented in parallel to the person's hidden right hand in an anatomically plausible position, and the touches delivered to the two hands in view had to be synchronous. These observations suggest that visual, tactile and proprioceptive information from the two hands is integrated automatically, even in the absence of bimanual action or bimanual tactile exploration, and that this bilateral multisensory integration can cause qualitative changes in tactile perception and limb ownership.

The questionnaire ratings revealed that only 16 out of the 30 participants, equivalent to 53%, reported feeling the illusion at all (gave scores of 1 or higher to statement S1). This is less than the original rubber-hand illusion which is perceived by approximately 70% of the participants [26], [32], [39]. Furthermore, the illusion presented here requires a longer period of stimulation to be elicited (typically minutes), while the original rubber hand illusion is experienced in most of the cases after only ten to fifteen seconds of synchronized brushing [26], [32]. These differences suggest that the bilateral transfer illusion requires that additional processes related to the integration of visual and tactile input from the opposite sides of the body be implicated.

It is important to emphasize that our data rule out the possibility that the present perceptual effect is merely a weak rubber hand illusion as described by [32] who demonstrated that the greater the distance between the rubber hand and the participant's real hand, the weaker the illusion. In this study the distance over which referred tactile sensations can be attributed to an artificial hand from the same laterality was estimated to be approximately a maximum of thirty centimeters [32]. In our set-up, the distance between the stimulated left hand and the right rubber hand was forty-two centimeters, which according to Lloyd's data would suggest that the illusion would not work very well. Crucially, in our fourth experiment, we made a direct comparison of the difference in the illusions when a right or a left rubber hand was brushed in synchrony with the left hand, the real right hand being hidden from view throughout. Importantly, only the right rubber hand produced a significant drift in proprioception, which demonstrates that the bilateral transfer illusion involves different processes.

Along the same argument, it is also unlikely that the present bilateral transfer illusion relies on the same process that created the duplication of touch sensation onto two rubber hands in the recently described “three-arm illusion” [40]. In this experiment, the person's right hand is placed under a table and two right rubber hands are placed side by side (10 cm apart), 10 cm above the real hand. Simultaneous brushstrokes applied to the three hands produced the sensation of touch on both rubber hands. However, for this illusion to work, the rubber hands have to be of the same laterality as the stimulated real hand (as found in the pilot experiments). Importantly, in the fourth of our experiments reported here, the left rubber hand condition effectively served as a control for a putative duplication of touch from the brushed left hand to *any* rubber hand placed 42 cm to the right of the stimulated hand. We observed a significantly greater proprioceptive drift in the right hand condition, eliciting the bilateral transfer illusion.

To the best of our knowledge, the present illusion is the first where tactile sensations are transferred from one limb to another across the body midline in healthy participants. In the ‘cutaneous rabbit illusion’ rapid stimulation at the wrist followed by stimulation near the elbow creates the illusory perception of touch at intervening locations along the arm [41], [42]. In another illusion, the so-called “tactile funnelling illusion”, people experience one touch at a location between two close sites of physical stimulation on the skin [43], [44], [45], [46]. In the somatosensory version of Sham's “double flash illusion” [47], participants experience two brief touches when the index finger is tapped once in combination with two brief flashes or auditory clicks [48]. All of these illusions are associated with a shift in the perceived location of touch on a limb, or with the duplication of the number of touches experienced at a particular location. The illusion reported here, however, is different because the touch was transferred between two homologous limbs. Thus, a right rubber hand ‘felt’ the touch that was applied to the left hand.

What brain mechanisms might be responsible for the present bilateral illusion? The transfer of tactile information from the left to the right hand could be mediated by neurons with bilateral tactile receptive fields in the parietal cortex. Electrophysiological studies in primates have revealed a substantial number of neurons with bilateral tactile receptive fields in Brodmann's areas 2 and 5 [16], [17], [18]. Such cells probably exist in the human brain too, as fMRI experiments have reported ipsilateral activation in areas 2 and 5 during unilateral somatosensory stimulation of the hand [49], [50]. Similarly, in non-human primates, cells with bilateral tactile receptive fields have been found in the parietal operculum in areas neighboring the SII cortex [51], [52]. Positron emission tomography [53], [54], fMRI [55] and magnetoencephalograpy [56], too, show bilateral responses in the parietal operculum in humans during unilateral tactile stimulation. The ipsilateral tactile responses in primates are likely mediated by callosal projections from the contralateral somatosensory areas, although thalamocortical input from the ventrobasal complex is a viable alternative [57]. Humans who have their corpus callosum sectioned as part of surgical procedures show reduced or eliminated ipsilateral responses in SII and parietal areas 2 and 5 [58]. Iwamura demonstrated that lesions of the contralateral SI in monkeys eliminated most of the ipsilateral responses in areas 2 and 5, which is consistent with the interpretation that these cells receive tactile information from the contralateral somatosensory cortex via callosal connections [17].

Thus, a plausible scenario for the ‘phantom touch’ reported here would be that the prolonged tactile stimulation of the participant's left hand generated weak activation in ipsilateral somatosensory areas. The time which was necessary for the ‘phantom touch’ to be perceived suggests that, initially, these ipsilateral responses were below the ‘threshold for conscious perception’. However, when the sub-threshold ipsilateral activation was combined with the temporally and spatially congruent visual information from the contralateral rubber hand, the ipsilateral tactile responses were up-regulated and produced the ‘phantom’ touch sensations. The visual information from the brushed right rubber hand could influence the ipsilateral tactile processing at several cortical nodes in the left hemisphere. Although areas 2, 5, and SII do not receive strong visual input [but see [20], [59]], they are reciprocally connected to multisensory areas such as the ventral premotor cortex [60], [61], [62], area 7 in the inferior parietal cortex [51], [52], [63], [64], and the ventral intraparietal area [VIP; [65], [66], [67]], all of which are known to be areas that receive substantial visual input [68], [69], [70], [71]. Thus, within these fronto-parietal circuits, the ipsilateral tactile information could be fused with visual and proprioceptive information from the right hand. This could be achieved by the integration of visual and tactile signals in arm-centered reference frames centered on the right rubber hand [27], implemented by neuronal populations in the ventral premotor cortex and the intraparietal cortex [26], [39].

It is still an open question whether the bimanual transfer of touch in healthy individuals involves similar mechanisms to those that induce ‘phantom touch’ sensations in patients with hemisensory loss or amputation [12], [13], [15]. In these cases, subsequent to brain damage or the loss of a limb, central plasticity could lead to a strengthening of the commissural connections and the ipsilateral somatosensory representations. In healthy individuals, as used in the case of our study, it seems that a couple of minutes of congruent visual and tactile stimulation is sufficient to up-regulate the ipsilateral somatosensory processing. Future imaging experiments are needed to characterize this hypothesized up-regulation process and to localize the neural correlates of the phantom touches with precision.

In conclusion, our study has introduced a novel version of the rubber-hand illusion in which converging multisensory input from both sides of the body suffices to change the feeling of limb ownership and to elicit illusory tactile sensations on an un-stimulated limb. This reveals an inter-hemispheric mechanism for tactile perception and multisensory integration which is involved in the perception of our own bodies. Our finding could have a bearing on applied neuroscience, as tactile stimulation to an intact hand in amputees might support the ownership and usage of prosthetic limbs [31], [72]. Similarly, research on stroke rehabilitation should examine the possibility that physiotherapy of a hemiplegic limb might be facilitated by concurrent tactile stimulation of the contralateral limb.

## References

[1] Press C, Taylor-Clarke M, Kennett S, Haggard P (2004). Visual enhancement of touch in spatial body representation.. Experimental Brain Research.

[2] Tipper S, Phillips N, Dancer C, Lloyd D, Howard L (2001). Vision influences tactile perception at body sites that cannot be viewed directly.. Experimental Brain Research.

[3] Ernst MO, Banks MS (2002). Humans integrate visual and haptic information in a statistically optimal fashion.. Nature.

[4] Lederman S, Abbott S (1981). Texture perception: studies of intersensory organization using a discrepancy paradigm, and visual versus tactual psychophysics.. J Exp Psychol Hum Percept Perform.

[5] Rock I, Victor J (1964). Vision and Touch: An Experimentally Created Conflict between the Two Senses.. Science.

[6] Haggard P, Christakou A, Serino A (2007). Viewing the body modulates tactile receptive fields.. Experimental Brain Research.

[7] Kennett S, Taylor-Clarke M, Haggard P (2001). Noninformative vision improves the spatial resolution of touch in humans.. Current Biology.

[8] Driver J, Noesselt T (2008). Multisensory Interplay Reveals Crossmodal Influences on ‘Sensory-Specific’ Brain Regions, Neural Responses, and Judgments.. Neuron.

[9] Lakatos P, Chen C-M, O'Connell MN, Mills A, Schroeder CE (2007). Neuronal Oscillations and Multisensory Interaction in Primary Auditory Cortex.. Neuron.

[10] Shimojo S, Shams L (2001). Sensory modalities are not separate modalities: plasticity and interactions.. Current Opinion in Neurobiology.

[11] Stein BE, Stanford TR (2008). Multisensory integration: current issues from the perspective of the single neuron.. Nat Rev Neurosci.

[12] Halligan P, Hunt M, Marshall J, Wade T (1996). When seeing is feeling: Acquired synaesthesia or phantom touch?. Neurocase.

[13] Sathian K (2008). Intermanual referral of sensation to anesthetic hands.. Neurology.

[14] Ramachandran VS, Rogers-Ramachandran D (1996). Synaesthesia in Phantom Limbs Induced with Mirrors.. Proc R Soc Lond B.

[15] Ramachandran VS, Rogers-Ramachandran D, Cobb S (1995). Touching the phantom limb.. Nature.

[16] Iwamura Y (2000). Bilateral receptive field neurons and callosal connections in the somatosensory cortex.. Philos Trans R Soc Lond B Biol Sci.

[17] Iwamura Y, Iriki A, Tanaka M (1994). Bilateral hand representation in the postcentral somatosensory cortex.. Nature.

[18] Iwamura Y, Tanaka M, Iriki A, Taoka M, Toda T (2002). Processing of tactile and kinesthetic signals from bilateral sides of the body in the postcentral gyrus of awake monkeys.. Behavioural Brain Research.

[19] Colby CL, Duhamel J-R (1991). Heterogeneity of extrastriate visual areas and multiple parietal areas in the Macaque monkey.. Neuropsychologia.

[20] Graziano MSA, Cooke DF, Taylor CSR (2000). Coding the Location of the Arm by Sight.. Science.

[21] Johnson MJ, Alloway KD (1996). Cross-correlation analysis reveals laminar differences in thalamocortical interactions in the somatosensory system.. J Neurophysiol.

[22] Jones E, Coulter J, Hendry S (1978). Intracortical connectivity of architectonic fields in the somatic sensory, motor and parietal cortex of monkeys.. The Journal of comparative neurology.

[23] Rizzolatti G, Luppino G, Matelli M (1998). The organization of the cortical motor system: new concepts.. Electroencephalogr Clin Neurophysiol.

[24] Sakata H, Takaoka Y, Kawarasaki A, Shibutani H (1973). Somatosensory properties of neurons in the superior parietal cortex (area 5) of the rhesus monkey.. Brain Research.

[25] Botvinick M, Cohen J (1998). Rubber hands ‘feel’ touch that eyes see.. Nature.

[26] Ehrsson HH, Spence C, Passingham RE (2004). That's My Hand! Activity in Premotor Cortex Reflects Feeling of Ownership of a Limb.. Science.

[27] Makin TR, Holmes NP, Ehrsson HH (2008). On the other hand: Dummy hands and peripersonal space.. Behavioural Brain Research.

[28] Tsakiris M, Haggard P (2005). The Rubber Hand Illusion Revisited: Visuotactile Integration and Self-Attribution.. Journal of Experimental Psychology: Human Perception and Performance.

[29] Costantini M, Haggard P (2007). The rubber hand illusion: Sensitivity and reference frame for body ownership.. Consciousness and Cognition.

[30] Pavani F, Spence C, Driver J (2000). Visual Capture Of Touch: Out-of-the-Body Experiences With Rubber Gloves.. Psychological Science.

[31] Ehrsson HH, Rosen B, Stockselius A, Ragno C, Kohler P (2008). Upper limb amputees can be induced to experience a rubber hand as their own.. Brain.

[32] Lloyd DM (2007). Spatial limits on referred touch to an alien limb may reflect boundaries of visuo-tactile peripersonal space surrounding the hand.. Brain and Cognition.

[33] Botvinick M (2004). Neuroscience. Probing the neural basis of body ownership.. Science.

[34] Graziano MSA (1999). Where is my arm? The relative role of vision and proprioception in the neuronal representation of limb position.. Proceedings of the National Academy of Sciences of the United States of America.

[35] McCabe CS, Haigh RC, Halligan PW, Blake DR (2005). Simulating sensory-motor incongruence in healthy volunteers: implications for a cortical model of pain.. Rheumatology.

[36] Armel KC, Ramachandran VS (2003). Projecting sensations to external objects: evidence from skin conductance response.. Proceedings of the Royal Society B: Biological Sciences.

[37] Ehrsson HH, Wiech K, Weiskopf N, Dolan RJ, Passingham RE (2007). Threatening a rubber hand that you feel is yours elicits a cortical anxiety response.. Proceedings of the National Academy of Sciences.

[38] Dawson M, Schell A, Filion D, Cacioppo J, Tassinary L, Berntson G (2007). The electrodermal system.. The handbook of psychophysiology.

[39] Ehrsson HH, Holmes NP, Passingham RE (2005). Touching a Rubber Hand: Feeling of Body Ownership Is Associated with Activity in Multisensory Brain Areas.. J Neurosci.

[40] Ehrsson HH (2009). How many arms make a pair? Perceptual illusion of having an additional arm.. Perception.

[41] Blankenburg F, Ruff CC, Deichmann R, Rees G, Driver J (2006). The Cutaneous Rabbit Illusion Affects Human Primary Sensory Cortex Somatotopically.. PLoS Biology.

[42] Geldard FA, Sherrick CE (1972). The Cutaneous “Rabbit”: A Perceptual Illusion.. Science.

[43] Chen L, Friedman R, Roe A (2003). Optical Imaging of a Tactile Illusion in Area 3b of the Primary Somatosensory Cortex.. Science.

[44] Gardner EP, Costanzo RM (1980). Spatial integration of multiple-point stimuli in primary somatosensory cortical receptive fields of alert monkeys.. J Neurophysiol.

[45] Sherrick CE (1964). Effects of double simultaneous stimulations of the skin.. The American journal of psychology.

[46] von Békésy G (1967). Sensory Inhibition..

[47] Shams L, Kamitani Y, Shimojo S (2000). Illusions: What you see is what you hear.. Nature.

[48] Wozny DR, Beierholm UR, Shams L (2008). Human trimodal perception follows optimal statistical inference.. Journal of Vision.

[49] Hlushchuk Y, Hari R (2006). Transient Suppression of Ipsilateral Primary Somatosensory Cortex during Tactile Finger Stimulation.. J Neurosci.

[50] Nihashi T, Naganawa S, Sato C, Kawai H, Nakamura T (2005). Contralateral and ipsilateral responses in primary somatosensory cortex following electrical median nerve stimulation–an fMRI study.. Clinical Neurophysiology.

[51] Robinson C, Burton H (1980). Organization of somatosensory receptive fields in cortical areas 7b, retroinsula, postauditory and granular insula of M. fascicularis.. The Journal of comparative neurology.

[52] Robinson C, Burton H (1980). Somatotopographic organization in the second somatosensory area of M. fascicularis.. The Journal of comparative neurology.

[53] Burton H, Videen T, Raichle M (1993). Tactile-vibration-activated foci in insular and parietal-opercular cortex studied with positron emission tomography: mapping the second somatosensory area in humans.. Somatosens Mot Res.

[54] Ledberg A, O'Sullivan B, Kinomura S, Roland P (1995). Somatosensory activations of the parietal operculum of man. A PET study.. Eur J Neurosci.

[55] Ruben J, Schwiemann J, Deuchert M, Meyer R, Krause T (2001). Somatotopic organization of human secondary somatosensory cortex.. Cerebral Cortex.

[56] Simões C, Hari R (1999). Relationship between Responses to Contra- and Ipsilateral Stimuli in the Human Second Somatosensory Cortex SII.. NeuroImage.

[57] Iwamura Y, Taoka M, Iriki A (2001). Bilateral activity and callosal connections in the somatosensory cortex.. Neuroscientist.

[58] Fabri M, Polonara G, Quattrini A, Salvolini U, Del Pesce M (1999). Role of the corpus callosum in the somatosensory activation of the ipsilateral cerebral cortex: an fMRI study of callosotomized patients.. Eur J Neurosci.

[59] Iriki A, Tanaka M, Iwamura Y (1996). Coding of modified body schema during tool use by macaque postcentral neurones.. Neuroreport.

[60] Gentilucci M, Fogassi L, Luppino G, Matelli M, Camarda R (1988). Functional organization of inferior area 6 in the macaque monkey. I. Somatotopy and the control of proximal movements.. Experimental Brain Research.

[61] Graziano MSA, Hu XT, Gross CG (1997). Visuospatial Properties of Ventral Premotor Cortex.. J Neurophysiol.

[62] Rizzolatti G, Scandolara C, Matelli M, Gentilucci M (1981). Afferent properties of periarcuate neurons in macaque monkeys. I. Somatosensory responses.. Behavioural Brain Research.

[63] Duhamel J-R, Bremmer F, BenHamed S, Graf W (1997). Spatial invariance of visual receptive fields in parietal cortex neurons.. Nature.

[64] Hyvarinen J (1981). Regional distribution of functions in parietal association area 7 of the monkey.. Brain Research.

[65] Avillac M, Ben Hamed S, Duhamel J-R (2007). Multisensory Integration in the Ventral Intraparietal Area of the Macaque Monkey.. J Neurosci.

[66] Colby CL, Duhamel JR, Goldberg ME (1993). Ventral intraparietal area of the macaque: anatomic location and visual response properties.. J Neurophysiol.

[67] Duhamel J-R, Colby CL, Goldberg ME (1998). Ventral Intraparietal Area of the Macaque: Congruent Visual and Somatic Response Properties.. J Neurophysiol.

[68] Godschalk M, Lemon R, Kuypers H, Ronday H (1984). Cortical afferents and efferents of monkey postarcuate area: an anatomical and electrophysiological study.. Experimental Brain Research.

[69] Matelli M, Camarda R, Glickstein M, Rizzolatti G (1986). Afferent and efferent projections of the inferior area 6 in the macaque monkey.. The Journal of comparative neurology.

[70] Neal JW, Pearson RCA, Powell TPS (1987). The cortico-cortical connections of area 7b, PF, in the parietal lobe of the monkey.. Brain Research.

[71] Rizzolatti G, Luppino G (2001). The Cortical Motor System.. Neuron.

[72] Rosén B, Ehrsson HH, Antfolk C, Cipriani C, Sebelius F (2009). Referral of sensation to an advanced humanoid robotic hand prosthesis.. Scand J Plast Rec Surg Hand Surg in press.

